# Post-Pubertal Deepbite Changes in Patients With or Without Previous Early Interceptive Treatment: A Retrospective Observational Study

**DOI:** 10.3390/jcm15208153

**Published:** 2026-10-20

**Authors:** Irene Lomonaco, Francesco Caroccia, Michele Nieri, Veronica Giuntini, Lorenzo Franchi

**Affiliations:** Department of Experimental and Clinical Medicine, University of Florence, 50134 Florence, Italy; irene.lomonaco@unifi.it (I.L.); michelenieri@gmail.com (M.N.); veronica.giuntini@unifi.it (V.G.); lorenzo.franchi@unifi.it (L.F.)

**Keywords:** deepbite, treatment timing, two-phase treatment, one-phase treatment, fixed appliances, orthodontic outcomes

## Abstract

**Objectives:** To compare overbite changes from the beginning of fixed appliance treatment to the post-pubertal assessment during retention between patients who had previously undergone early interceptive treatment and patients treated without a previous interceptive phase. **Methods:** This retrospective longitudinal study included 34 patients (13 females and 21 males) with an initial overbite ≥4.5 mm. Seventeen patients had undergone an early interceptive phase (ETG), whereas 17 received fixed appliance treatment without previous interceptive treatment (LTG). T0 corresponded to the beginning of fixed appliance treatment in both groups; in the ETG, T0 followed Phase 1 treatment and an intermediate retention period. T1 was obtained during posttreatment retention at a post-pubertal stage. Overbite was the primary outcome, while the remaining skeletal and dentoalveolar measurements were considered secondary exploratory outcomes. T1–T0 changes were compared between groups using ANCOVA with the corresponding T0 value as a covariate. **Results:** At T0, the ETG showed a smaller overbite (4.1 ± 1.2 mm vs. 5.6 ± 0.9 mm) and a greater U1–PP angle (111.5° ± 5.1° vs. 104.8° ± 6.7°). During T0–T1, overbite decreased by 0.8 ± 1.0 mm in the ETG and 2.5 ± 1.6 mm in the LTG. After accounting for the corresponding T0 value, the between-group difference in overbite change was 0.8 mm (95% CI, −0.2 to 1.8; *p* = 0.118). No statistically significant between-group difference in overbite change was detected. No statistically significant between-group differences were detected for the secondary skeletal or dentoalveolar outcomes. **Conclusions:** Within the limitations of this retrospective study, no statistically significant between-group difference in overbite change was detected. However, the study had limited power to exclude a clinically meaningful difference, and the findings should not be interpreted as evidence of equivalence.

## 1. Introduction

Despite its high prevalence, the orthodontic management of deepbite has received relatively limited attention in the scientific literature [1,2]. One possible explanation is that deepbite is rarely considered an isolated malocclusion but is more commonly associated with sagittal discrepancies, particularly Class II division 1 and Class II division 2 malocclusions [3]. The defining characteristic of this condition is an increased overbite, which generally results from a combination of skeletal factors, such as a hypodivergent growth pattern, and dentoalveolar components, including incisor retroclination, incisor overeruption, an accentuated curve of Spee, and undereruption of the posterior teeth.

Several treatment approaches have been proposed to reduce excessive overbite, including anterior bite planes [4,5], continuous archwire mechanics [6], cervical headgear [7,8,9], utility arches [10], and temporary anchorage devices (TADs) [11]. Despite their widespread clinical use, evidence regarding the efficacy and long-term stability of deepbite correction remains limited [2]. Clinical studies specifically designed to evaluate treatment outcomes in patients with deepbite are relatively scarce, and many lack direct comparisons between different treatment approaches [12,13,14]. Available evidence indicates that orthodontic treatment can achieve moderate reductions in overbite; however, some degree of relapse is frequently observed during the posttreatment period [13,14].

Recent evidence has further highlighted the complexity of deepbite correction, particularly with respect to long-term stability. A systematic review by El Guennouni et al. [2], which included six clinical studies, reported heterogeneous outcomes according to treatment timing and modality. Early interceptive treatment during the mixed dentition showed inconsistent stability, with some studies reporting stable outcomes [15] and others demonstrating a significant increase in overbite after retention [16]. Conversely, treatment initiated during the late mixed or permanent dentition appeared to provide more consistent long-term stability [17,18]. In addition, specific treatment mechanics, such as incisor intrusion, have been associated with more favorable stability outcomes [19].

Another unresolved issue concerns the optimal timing of treatment. Two-phase therapy initiated during the mixed dentition has been advocated to intercept the developing malocclusion and potentially guide vertical dentoalveolar development by controlling incisor eruption while promoting relative posterior eruption [5,7,9]. Alternatively, a one-phase approach initiated during the pubertal growth spurt or after puberty, once the permanent dentition is established and the second molars have fully erupted, may represent an alternative treatment strategy [14]. However, definitive evidence regarding the influence of treatment timing on treatment efficacy and long-term stability remains lacking [12,14,20].

The aim of this retrospective longitudinal study was to compare overbite changes from the beginning of fixed appliance treatment to the post-pubertal assessment during retention between patients who had previously undergone early interceptive treatment and patients treated with fixed appliances without a previous interceptive phase. Secondary skeletal and dentoalveolar changes were also evaluated.

## 2. Materials and Methods

### 2.1. Study Design, Setting, and Participants

This retrospective longitudinal study was reported according to the Strengthening the Reporting of Observational Studies in Epidemiology (STROBE) guidelines [21] (Supplementary Table S1). Artificial intelligence (ChatGPT, OpenAI, San Francisco, CA, USA) was used exclusively to assist with English language editing during manuscript preparation. The study was conducted in accordance with the Declaration of Helsinki and was approved by the Regional Pediatric Ethics Committee of Tuscany (Florence, Italy; protocol LF20; approval no. 157/2025; date of approval: 15 July 2025).

Sample size was calculated to detect a between-group difference of 1.5 mm in the primary outcome variable (overbite). A standard deviation of 1.5 mm was derived from a previous study [13]. An alpha level of 0.05 and a statistical power of 80% were adopted, resulting in a required sample size of 17 subjects per group.

Lateral cephalograms of 34 Caucasian patients (13 females and 21 males) with deepbite malocclusion who had been consecutively treated with fixed appliances (FAs), with or without a preceding early interceptive phase (Phase 1), were retrospectively collected. The records were retrieved from patients treated at the Orthodontic Clinic of the Careggi University Hospital, Florence, Italy, between January 1996 and November 2022.

The inclusion criteria were the presence of deepbite malocclusion, defined as an overbite (OB) ≥ 4.5 mm on the initial lateral cephalogram, and fully erupted maxillary and mandibular incisors. In the ETG, initial OB and skeletal maturation were assessed on the lateral cephalogram obtained before Phase 1; this observation was not used as T0 for the T0–T1 comparison. For both groups, T0 was defined as the lateral cephalogram obtained immediately before the start of FA therapy, and T1 as the lateral cephalogram obtained during the retention period at a post-pubertal stage. Thus, although T0 represented the beginning of FA therapy in both groups, in the ETG it followed Phase 1 and an intermediate retention period, whereas in the LTG it represented the beginning of orthodontic treatment. The exclusion criteria were cleft lip and/or palate, craniofacial syndromes, and dental agenesis.

Patients were divided into two groups according to their treatment pathway. The early treatment group (ETG) comprised 17 subjects (8 females and 9 males) who underwent Phase 1 during the mixed dentition with either a removable maxillary plate incorporating an anterior bite plane (10 subjects) or cervical headgear combined with an anterior bite plane (7 subjects), followed by Phase 2 with FAs in the permanent dentition. The 17 ETG subjects included in the present study were drawn from the early-treatment cohort previously investigated by Baccetti et al. [14]. Accordingly, the present investigation represents a secondary analysis involving this previously studied ETG cohort combined with a new late-treatment comparison group. Before Phase 1, all ETG subjects were at a prepubertal stage of skeletal maturation (CS1 or CS2) according to the Cervical Vertebral Maturation (CVM) method [22]. At T0, immediately before FA therapy, all ETG subjects had reached a pubertal or post-pubertal stage (CS3, CS4, or CS5). The late treatment group (LTG) comprised 17 subjects (5 females and 12 males) who had received no previous interceptive treatment and underwent FA treatment in the permanent dentition after complete eruption of the second molars. At T0, all LTG subjects were also at a pubertal or post-pubertal stage (CS3, CS4, or CS5).

Fixed appliance therapy followed the same standardized protocol in both groups using 0.022-inch slot preadjusted edgewise appliances with an MBT prescription. The archwire sequence consisted of 0.014-inch and 0.016-inch round nickel-titanium (NiTi) archwires, followed by 0.019 × 0.025-inch rectangular nickel-titanium archwires and subsequently 0.019 × 0.025-inch stainless steel archwires with soldered posts. Uniform arch forms were used throughout treatment, and brackets were bonded on all teeth from the second molar to the contralateral second molar. All subjects in both groups underwent non-extraction treatment. In both groups, fixed auxiliaries for deepbite correction, including a modified Nance button and anterior bite ramps, could be used when clinically indicated. Retention protocols differed between groups. During the intermediate retention period between Phase 1 and FA therapy, ETG patients wore a removable maxillary plate incorporating an anterior bite plane. After FA therapy, ETG patients were retained with Hawley retainers in both arches, whereas LTG patients were retained with removable Essix retainers in both arches. T1 cephalograms were obtained during the retention period, with removable retainers temporarily removed immediately before radiographic acquisition.

### 2.2. Cephalometric Analysis

**Figure 1:**
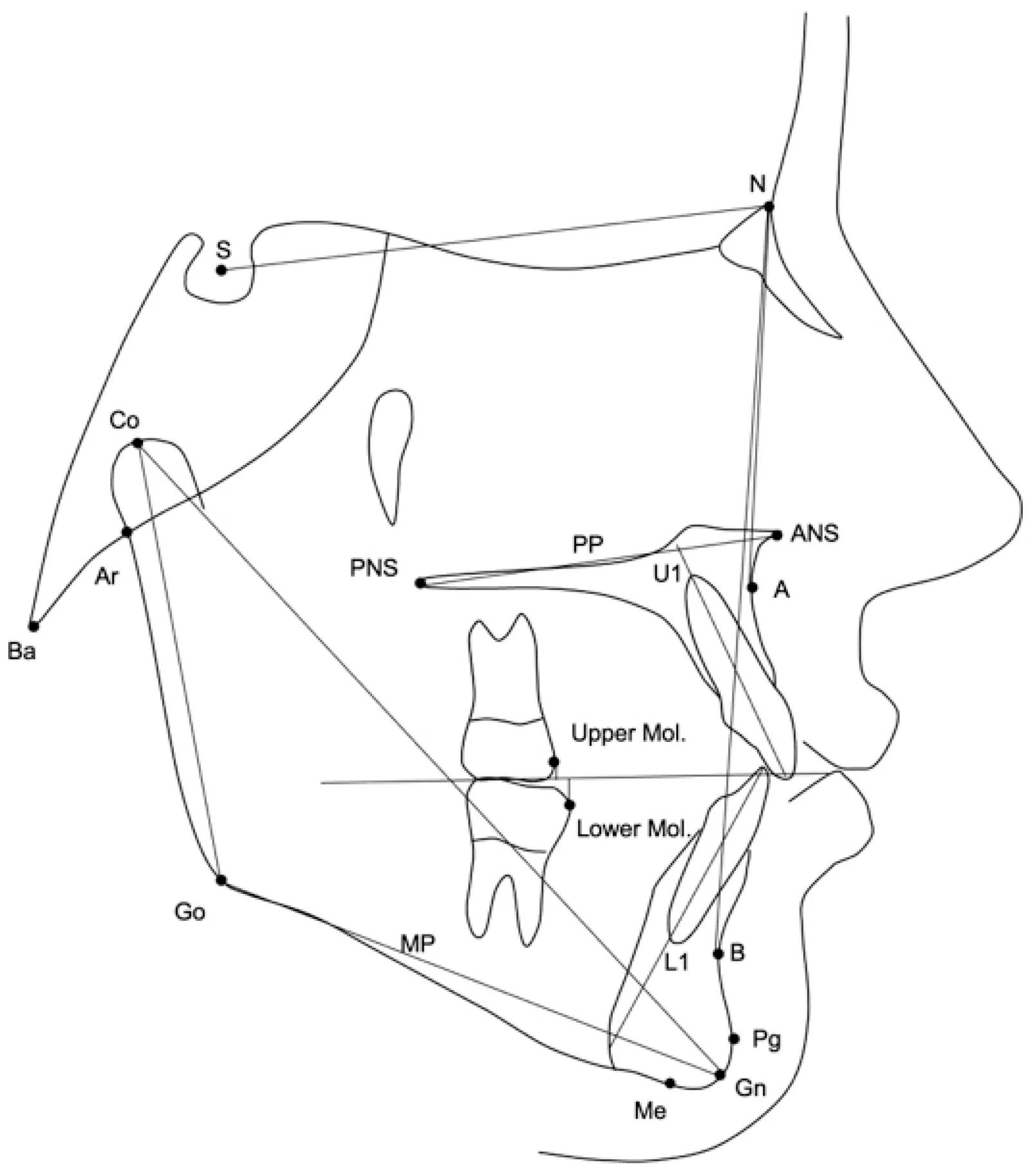
Cephalometric landmarks and planes: S, Sella; N, Nasion; Ba, Basion; PNS, Posterior Nasal Spine; ANS, Anterior Nasal Spine; PP, Palatal Plane; MP, Mandibular Plane; Go, Gonion; Co, Condylion; Me, Menton; Gn, Gnathion; A, A point; B, B point; Upper Mol., most mesial point of the upper first molar; Lower Mol., most mesial point of the lower first molar, U1, axis of the upper incisor; L1, axis of the lower incisor; Pg, pogonion; Ar, Articulare.

Cephalometric tracings were performed by a single investigator (IL). All cephalograms were digitized using a customized cephalometric analysis with Viewbox 4.0 software (dHAL Software, Kifissia, Athens, Greece). Radiographic magnification was standardized at 0%. The cephalometric variables included 10 angular measurements: NSBa, SNA, SNB, ANB, SN-Palatal Plane (SN-PP), SN-Mandibular Plane (SN-MP), Palatal Plane–Mandibular Plane (PP-MP), CoGoMe, Upper Incisor–Palatal Plane (U1-PP), and Lower Incisor–Mandibular Plane (L1-MP), together with 8 linear measurements: Wits appraisal (the linear distance along the occlusal plane between the projections on the functional occlusal plane of points A and B. This measure is positive if Point A is ahead of Point B), Co-Gn, Co-Go, N-Me, ANS-Me, molar relationship (distance between the projections of upper first molar and lower first molar on the occlusal plane), overjet (distance measured parallel to the functional occlusal plane from the incisal edge of the most protrusive maxillary central incisor to the most facial aspect of the incisal third of the crown of the most protrusive mandibular central incisor), and overbite (distance measured perpendicular to the functional occlusal plane between the incisal edges of the most protrusive maxillary and mandibular central incisors). The functional occlusal plane is drawn through the occlusal contacts of the first premolars (or first deciduous molars) and of the first permanent molars (Figure 1).

### 2.3. Method Error

Fifteen cephalograms were randomly selected from the total sample and re-digitized by the same investigator (IL) after a 2-week washout period. Intra-examiner agreement for CVM staging, an ordinal variable, was assessed using weighted Cohen’s kappa with quadratic weights. Intra-examiner reliability for the continuous cephalometric measurements was assessed using a two-way mixed-effects intraclass correlation coefficient (ICC) model for absolute agreement, based on single measurements.

### 2.4. Statistical Analysis

Descriptive statistics were calculated as means and standard deviations for quantitative variables and as frequencies and percentages for categorical variables. Independent-samples t tests were used to compare age and all cephalometric variables between the two groups at T0. Overbite was defined a priori as the primary outcome; all other skeletal and dentoalveolar cephalometric measurements were considered secondary exploratory outcomes. Treatment changes (T1–T0) were analyzed using analysis of covariance (ANCOVA), with the corresponding T0 value entered as a covariate.

The assumptions of ANCOVA were assessed for all cephalometric variables. Homogeneity of regression slopes was evaluated by testing the interaction between treatment group and the corresponding T0 value. Normality of residuals was assessed by visual inspection of Q–Q plots and the Shapiro–Wilk test, whereas homoscedasticity was evaluated using residuals-versus-fitted plots and the Breusch–Pagan test. For models showing potential assumption violations, sensitivity analyses were performed using heteroscedasticity-consistent HC3 robust standard errors.

Differences in gender distribution between groups were assessed using Fisher’s exact test.

All statistical analyses were performed using IBM SPSS Statistics (version 31.0; IBM Corp., Armonk, NY, USA) and MedCalc (version 19.6.4; MedCalc Software Ltd., Ostend, Belgium).

## 3. Results

Weighted Cohen’s kappa for CVM staging was 0.87 (95% CI, 0.77–0.96), indicating almost perfect intra-examiner agreement [23]. For the continuous cephalometric measurements, ICC values exceeded 0.90 for almost all variables, indicating excellent reliability; the only exceptions were the ANB angle and Wits appraisal, which showed good reliability (ICC between 0.75 and 0.90) [24]. The corresponding reliability estimates and 95% confidence intervals are reported in Table 1.

Fisher’s exact test showed no statistically significant difference in gender distribution between the two groups (*p* = 0.481).

A total of 34 subjects were included in the study: 17 in the ETG (8 females and 9 males) and 17 in the LTG (5 females and 12 males). In the ETG, the mean duration of Phase 1 was 18 months, followed by an intermediate retention period of 10–18 months before FA therapy. Phase 2 with FA lasted a mean of approximately 33 months and was followed by posttreatment retention for approximately 12 months before T1. In the LTG, mean FA treatment duration was approximately 34 months, followed by a mean posttreatment retention period of approximately 9 months before T1.

At T0, no statistically significant between-group difference in age was observed (ETG: 12.6 ± 1.4 years; LTG: 13.1 ± 1.1 years; *p* = 0.319), and no statistically significant differences were detected for most skeletal and dentoalveolar variables (Table 2). However, statistically significant differences were observed for two dentoalveolar variables. Overbite was smaller in the ETG than in the LTG (4.1 ± 1.2 mm vs. 5.6 ± 0.9 mm, respectively; mean difference −1.5 mm; 95% CI, −2.3 to −0.8 mm; *p* < 0.001). In addition, the U1-PP angle was greater in the ETG than in the LTG (111.5° ± 5.1° vs. 104.8° ± 6.7°, respectively; mean difference 6.7°; 95% CI, 2.6° to 10.9°; *p* = 0.002).

At T1, the mean age was 16.3 ± 2.0 years in the ETG and 16.7 ± 1.2 years in the LTG, and all subjects had reached a post-pubertal stage of skeletal maturation (CS5–CS6). For the primary outcome, overbite decreased by 0.8 ± 1.0 mm in the ETG and by 2.5 ± 1.6 mm in the LTG. The between-group difference was 0.8 mm (95% CI, −0.2 to 1.8; *p* = 0.118). No statistically significant between-group differences were detected for the secondary exploratory skeletal or dentoalveolar outcomes (Table 3). For U1-PP, the between-group difference was −4.0° (95% CI, −8.2° to 0.3°; *p* = 0.070).

For the primary outcome, no significant departures from residual normality (Shapiro–Wilk *p* = 0.105) or homoscedasticity (Breusch–Pagan *p* = 0.222) were detected, and the group-by-T0 interaction was not statistically significant (*p* = 0.165). Among the secondary outcomes, a potential violation of residual normality was identified for molar relationship (Shapiro–Wilk *p* = 0.006). Sensitivity analysis using HC3 robust standard errors confirmed the original finding, with no significant between-group difference for molar relationship (*p* = 0.718).

## 4. Discussion

The present retrospective longitudinal study evaluated T0–T1 changes from the beginning of fixed appliance therapy to the post-pubertal assessment during posttreatment retention in patients who had previously undergone early interceptive treatment and in patients treated with fixed appliances without a previous interceptive phase. T0 corresponded to the beginning of FA therapy in both groups; however, it represented different points within the overall treatment pathways because ETG patients had already completed Phase 1 and an intermediate retention period. Accordingly, the present analysis should not be interpreted as a direct comparison of the total effects of complete two-phase and one-phase treatment pathways.

For the primary outcome, no statistically significant between-group difference in overbite change was detected during the T0–T1 interval. The between-group estimate was 0.8 mm, with a 95% confidence interval from −0.2 to 1.8 mm. Because this study was designed as a superiority study rather than an equivalence or non-inferiority study, the absence of statistical significance should not be interpreted as evidence that the two treatment pathways are equivalent. Moreover, the confidence interval includes differences that may be clinically relevant, and the study had limited power to detect a between-group difference in the magnitude observed. No statistically significant between-group differences were detected for the secondary exploratory skeletal or dentoalveolar outcomes.

The differences observed in overbite and U1-PP at T0 are consistent with completion of the interceptive phase before initiation of FA therapy in the ETG. The smaller overbite and greater maxillary incisor proclination observed in the ETG at T0 are likely related, at least in part, to the preceding treatment. Although the corresponding T0 value was included as a covariate in the ANCOVA, residual confounding and regression-to-the-mean effects cannot be completely excluded in this retrospective sample.

The influence of treatment timing on deepbite correction remains controversial. A recent systematic review by El Guennouni et al. [2] concluded that treatment initiated in the permanent dentition was associated with greater efficacy and improved long-term stability than early interceptive treatment. However, two of the studies included in that review evaluated patients with Class II malocclusion, potentially limiting the applicability of those findings to patients presenting with deepbite irrespective of sagittal relationship. Furthermore, the delayed treatment protocol investigated in the present study differed from those included in the review, as treatment in the LTG was initiated after complete eruption of the second molars. This timing allows for incorporation of the second molars into the mechanics for levelling the curve of Spee and may facilitate posterior vertical development during treatment.

Previous longitudinal investigations have shown that untreated growing subjects may exhibit a spontaneous reduction in overbite resulting from continued eruption of the posterior teeth and vertical growth of the mandibular ramus [3,25]. In the absence of an untreated control group in the present study, the observed T1–T0 changes reflect the combined effects of orthodontic treatment and normal craniofacial development, and the relative contribution of these two components cannot be determined. This is particularly relevant when interpreting overbite change during growth.

Studies evaluating treatment initiated at later developmental stages have generally reported favorable long-term stability [17,18].

From a practical perspective, avoiding an additional interceptive phase and intermediate retention period may represent a potential advantage of a one-phase treatment pathway; however, treatment efficiency was not formally evaluated.

The present study shares part of its sample with the previous investigation by Baccetti et al. [14], as the 17 subjects in the ETG were drawn from the early-treatment cohort evaluated in that study. Because the ETG had been investigated previously, the present dataset is not fully independent of the earlier publication; however, the comparison group, observation interval, and clinical question are different. In the previous study, the late-treatment group also underwent an initial phase of deepbite treatment with an anterior bite plane, initiated at a pubertal stage and immediately followed by fixed appliance therapy. In contrast, the LTG in the present study consisted of patients who had received no previous interceptive treatment and started comprehensive fixed appliance therapy in the permanent dentition. Furthermore, whereas the previous investigation evaluated changes during the initial bite-plane phase, the fixed appliance phase, and the overall treatment period, the present study evaluated changes from the beginning of fixed appliance therapy (T0) to the post-pubertal T1 assessment. Although several cephalometric outcomes overlapped between the two investigations, including overbite and dentoskeletal measurements, the present study addressed a different clinical question: whether previous early interceptive treatment was associated with different changes from the beginning of fixed appliance therapy to the post-pubertal assessment during retention compared with patients who had received no previous interceptive treatment.

Several limitations of the present study should be acknowledged. First, treatment allocation was retrospective and non-randomized, and the reasons for choosing early or late treatment could not be reliably reconstructed from the clinical records. Consequently, confounding by indication cannot be excluded because factors influencing the decision to initiate early treatment may also have been associated with treatment outcomes. Second, although T0 corresponded to the beginning of fixed appliance therapy in both groups, it represented different points within the overall treatment pathways: in the ETG, T0 followed Phase 1 and an intermediate retention period, whereas in the LTG it represented the beginning of orthodontic treatment. Therefore, the T1-T0 comparison does not represent a comparison of the complete effects of the two treatment pathways.

The treatment pathways also differed in mean fixed appliance treatment duration and in the type and duration of posttreatment retention. Variation in retainer type and retention duration before T1 may have influenced posttreatment overbite stability and could therefore have confounded the between-group comparison. In addition, Phase 1 within the ETG was heterogeneous, because 10 patients were treated with an anterior bite plane alone and 7 with cervical headgear combined with an anterior bite plane; no subgroup sensitivity analysis was performed. These differences should be considered when interpreting the between-group findings. The relatively small sample size (17 subjects per group) also limited the precision of the estimates. The a priori sample-size calculation was based on detecting a 1.5 mm between-group difference in overbite, whereas the observed between-group difference was 0.8 mm and its 95% confidence interval was relatively wide. Thus, the study had limited power to detect a difference in the magnitude observed and cannot establish equivalence between the treatment pathways.

The recruitment period was long, spanning from January 1996 to November 2022, and the ETG was treated predominantly during the earlier years of this period. Patients in the two groups were not necessarily treated by the same clinicians. Although the fixed appliance protocol, archwire sequence, and approach to the use of auxiliaries remained unchanged in our clinic throughout the study period, potential changes in clinical practice over time and clinician-related variability cannot be excluded. The absence of an untreated control group is another important limitation; consequently, treatment-related changes cannot be separated from normal craniofacial growth. Finally, the inclusion of only Caucasian subjects may limit the generalizability of the findings.

Future prospective studies with larger samples and more comparable treatment pathways are needed to further clarify the association between previous early interceptive treatment and subsequent deepbite changes from the beginning of fixed appliance therapy to posttreatment retention.

## 5. Conclusions

Within the limitations of this retrospective study, no statistically significant between-group difference in overbite change during the T0–T1 observation interval was detected between patients who had previously undergone early interceptive treatment and those treated with fixed appliances without a previous interceptive phase. However, the study had limited power to exclude a clinically meaningful difference and does not provide evidence of equivalence between the two treatment pathways. No statistically significant between-group differences were detected for the secondary skeletal and dentoalveolar outcomes.

## Figures and Tables

**Table 1:** Intra-examiner agreement and reliability estimates for CVM staging (κw) and continuous cephalometric measurements (ICC).

Variables	Reliability Statistics	95% CI	
		Lower	Upper
NSBa (°)	0.98	0.94	0.99
SNA (°)	0.94	0.83	0.99
SNB (°)	0.99	0.97	1.00
ANB (°)	0.79	0.39	0.93
Wits appraisal (mm)	0.80	0.42	0.93
SN-PP (°)	0.98	0.94	0.99
SN-MP (°)	0.99	0.99	1.00
PP-MP (°)	0.99	0.99	1.00
Co-Gn (mm)	0.98	0.94	0.99
Co-Go (mm)	0.98	0.93	0.99
N-Me (mm)	0.99	0.99	1.00
ANS-Me (mm)	0.99	0.99	1.00
CoGoMe (°)	0.93	0.80	0.98
Overjet (mm)	0.98	0.95	0.99
Overbite (mm)	0.96	0.90	0.99
Molar Rel. (mm)	0.94	0.84	0.98
U1-PP (°)	0.99	0.96	1.00
L1-MP (°)	0.97	0.91	0.99
CVM stage (κw)	0.87	0.77	0.96

κw: weighted Cohen’s kappa; ICC: Intraclass Correlation Coefficient; CI: Confidence Interval; PP: Palatal Plane; MP: Mandibular Plane; Rel.: Relationship; U1: Upper incisor; L1: Lower incisor; CVM: Cervical Vertebral Maturation.

**Table 2:** Mean and standard deviation (SD) of the cephalometric variables at T0, differences between group means, *p* values, and corresponding 95% confidence intervals (CI).

	ETG n = 17 (8F; 9 M)		LTG n = 17 (5F; 12 M)				95% CI	
Variables	Mean	SD	Mean	SD	Mean Diff.	*p* Value	Lower	Upper
Age (years)	12.6	1.4	13.1	1.1	−0.5	0.319	−1.3	0.4
NSBa (°)	130.7	6.1	132.4	5.2	−1.7	0.395	−5.6	2.3
SNA (°)	80.7	1.7	81.9	3.4	−1.2	0.194	−3.1	0.7
SNB (°)	77.0	1.9	77.7	2.8	−0.7	0.374	−2.4	0.9
ANB (°)	3.7	1.9	4.2	2.1	−0.5	0.505	−1.9	0.9
Wits appraisal (mm)	0.4	3.8	0.8	3.5	−0.4	0.740	−3.0	2.1
SN-PP (°)	9.9	2.9	8.3	2.9	1.6	0.131	−0.5	3.6
SN-MP (°)	31.2	5.9	29.5	4.3	1.7	0.369	−2.0	5.3
PP-MP (°)	21.3	6.3	21.2	3.7	0.1	0.963	−3.5	3.7
Co-Gn (mm)	108.0	7.0	109.5	4.9	−1.5	0.486	−5.7	2.8
Co-Go (mm)	51.9	3.9	54.5	4.0	−2.6	0.057	−5.4	0.1
N-Me (mm)	112.1	6.4	110.1	4.8	2.0	0.313	−2.0	5.9
ANS-Me (mm)	61.1	5.2	61.5	4.3	−0.4	0.791	−3.8	2.9
CoGoMe (°)	122.6	5.9	122.2	4.0	0.4	0.839	−3.2	3.9
Overjet (mm)	3.7	1.4	3.1	1.5	0.6	0.228	−0.4	1.6
Overbite (mm)	4.1	1.2	5.6	0.9	−1.5	<0.001	−2.3	−0.8
Molar Rel. (mm)	1.4	1.6	0.2	1.7	1.2	0.050	0.0	2.4
U1-PP (°)	111.5	5.1	104.8	6.7	6.7	0.002	2.6	10.9
L1-MP (°)	92.7	6.1	96.9	7.0	−4.2	0.074	−8.8	0.4

PP: Palatal Plane; MP: Mandibular Plane; Rel.: Relationship; U1: Upper incisor; L1: Lower incisor; diff: difference; SD: Standard Deviation; CI: Confidence Interval.

**Table 3:** Mean and standard deviation (SD) of the T1–T0 changes in cephalometric variables, adjusted differences between group means, *p* values, and corresponding 95% confidence intervals (CI).

	ETG n = 17 (8F; 9 M)		LTG n = 17 (5F; 12 M)				95% CI	
Variables	Mean	SD	Mean	SD	Adjusted Mean Diff.	*p* Value	Lower	Upper
Age (years)	3.9	1.4	3.7	1.1	0.1	0.823	−0.8	1.0
NSBa (°)	0.3	2.3	0.6	2.3	−0.3	0.661	−2.0	1.3
SNA (°)	0.1	1.7	−0.9	1.2	0.9	0.105	−0.2	1.9
SNB (°)	0.3	1.6	0.4	1.6	0.0	0.963	−1.2	1.1
ANB (°)	−0.3	2.0	−1.4	2.1	1.0	0.162	−0.4	2.4
Wits appraisal (mm)	1.1	3.1	0.9	2.9	0.0	0.990	−1.8	1.8
SN-PP (°)	−0.1	1.3	−0.1	1.3	−0.1	0.806	−1.0	0.8
SN-MP (°)	−1.8	2.9	−1.3	2.2	−0.9	0.293	−2.5	0.8
PP-MP (°)	−1.7	2.8	−1.2	1.9	−0.5	0.535	−2.1	1.1
Co-Gn (mm)	5.7	4.8	6.1	4.8	−0.5	0.780	−3.9	3.0
Co-Go (mm)	4.5	4.7	4.7	3.7	−0.2	0.907	−3.4	3.0
N-Me (mm)	4.5	3.0	5.8	3.4	−1.1	0.353	−3.3	1.2
ANS-Me (mm)	2.9	1.8	3.7	2.1	−0.8	0.265	−2.1	0.6
CoGoMe (°)	−2.2	3.9	−2.1	1.7	−0.1	0.954	−2.2	2.1
Overjet (mm)	−0.5	1.8	−0.2	1.7	0.3	0.516	−0.5	1.1
Overbite (mm)	−0.8	1.0	−2.5	1.6	0.8	0.118	−0.2	1.8
Molar Rel. (mm)	−0.3	2.3	0.9	1.9	−0.2	0.737	−1.4	1.0
U1-PP (°)	1.2	4.2	9.2	7.9	−4.0	0.070	−8.2	0.3
L1-MP (°)	3.3	5.5	3.6	3.4	−1.5	0.338	−4.6	1.6

PP: Palatal Plane; MP: Mandibular Plane; Rel.: Relationship; U1: Upper incisor; L1: Lower incisor; diff: difference; SD: Standard Deviation; CI: Confidence Interval.

## Data Availability

The data presented in this study are available upon request from the corresponding authors.

## Supplementary Materials

The following supporting information can be downloaded at: https://www.mdpi.com/article/10.3390/jcm15208153/s1. Table S1: STROBE Statement—checklist of items that should be included in reports of observational studies.

## Abbreviations

The following abbreviations are used in this manuscript:

ETG	Early Treatment Group
LTG	Late Treatment Group
CVM	Cervical Vertebral Maturation
CS	Cervical Stage
FA	Fixed Appliances
